# Impact of Cold Ischemia on Mitochondrial Function in Porcine Hearts and Blood Vessels

**DOI:** 10.3390/ijms141122042

**Published:** 2013-11-07

**Authors:** Dominik Wiedemann, Thomas Schachner, Nikolaos Bonaros, Melissa Dorn, Martin Andreas, Alfred Kocher, Andrey V. Kuznetsov

**Affiliations:** 1 Department of Cardiac Surgery, Medical University of Vienna, Vienna A-1090, Austria; E-Mails: martin.andreas@meduniwien.ac.at (M.A.); alfred.kocher@meduniwien.ac.at (A.K.); 2 Cardiac Surgery Research Laboratory, Department of Cardiac Surgery, Innsbruck Medical University, Innsbruck A-6020, Austria; E-Mails: thomas.schachner@i-med.ac.at (T.S.); nikolaos.bonaros@i-med.ac.at (N.B.); andrej.kuznetsov@i-med.ac.at (A.V.K.); 3 Small Animal Surgery, Department of Small Animals and Horses, Veterinary Medicine University of Vienna, Vienna A-1210, Austria; E-Mail: melissa.dorn@vetmeduni.ac.at

**Keywords:** mitochonidria, respirometry, ischemia, preservation

## Abstract

The effects of cold storage using Custodiol^®^ (Histidine-Tryptophan-Ketoglutarate, HTK) or isotonic saline solution on mitochondrial function in hearts (left and rights ventricles) and various blood vessels of pigs were investigated. Hearts, saphenous veins, internal-mammary-arteries and aortas of male landrace pigs were harvested and exposed to cold ischemia in either saline or Custodiol-HTK solution. Mitochondrial function was measured *in situ* in permeabilized fibers by high-resolution respirometry. Mitochondrial respiratory capacities (maximal respiration rates) were similar in the right and left ventricle in controls and after 14 h of cold storage were significantly better preserved in Custodiol-HTK than in saline solution. Mitochondrial respiration rates in various blood vessels including aorta, arteries and veins were less than 5% of myocardium rates. In contrast to the pig heart, in some blood vessels, like veins, mitochondrial function remained stable even after 24 h of cold ischemia. HTK-Custodiol protection of mitochondrial function after prolonged cold ischemia was observed in the myocardium but not in blood vessels. HTK-Custodiol solution thus offers significant protection of myocardial mitochondria against cold ischemic injury and can be used as efficient preservation solution in organ transplantation but probably has no benefit for blood vessels preservation. Analysis of mitochondrial function can be used as a valuable approach for the assessment of cold ischemic injury in various tissues including pig heart and various blood vessels.

## 1. Introduction

Analysis of mitochondrial function has become central to basic research of mitochondrial physiology and the diagnosis of many diseases [1,2]. Assessment of mitochondrial oxidative phosphorylation is essential for studying cellular energetic processes and has become crucial in the assessment of neurodegenerative diseases, metabolic disorders, cancer, ageing and cardiovascular ischemia-reperfusion injury [3–6]. Mitochondrial respiratory function reflects the capacity for aerobic energy production of the heart, and is thus more indicative of organ viability than myocardial ATP levels [7–9]. Tissue ischemia causes cellular energy depletion, accumulation of toxic products like reactive oxygen species (ROS), mitochondrial failure and necrotic and apoptotic cell death [7,8]. Mitochondrial injury may also contribute to organ damage in acute and chronic rejection, causing a variety of subsequent deleterious effects [10]. A decline in mitochondrial respiratory capacity and diminished ability to generate ATP impairs energy metabolism during reperfusion and thereby contributes to irreversible cell membrane damage due to super-contracture [1]. Some pathologic stimuli may activate the inherent apoptotic program due to the ischemic stimulus, while other more pronounced cellular insults directly lead to cell death [9]. Furthermore, mitochondria play a central role in oxidative stress and control of cell death. Injured mitochondria are directly involved in the generation of reactive oxygen and nitrogen species during reperfusion (such as superoxide and peroxynitrite), inducing a cascade of pathological events [11]. Finally, release of mitochondrial cytochrome c is a key step in apoptosis resulting in cardiomyocyte death [7,9,11–13]. Cardiovascular surgery exposes the heart itself and also various blood vessels to prolonged periods of warm and cold ischemia. Especially in the heart transplantation, prolonged ischemia leads to higher rates of primary organ failure resulting in worse overall outcome of heart transplant recipients [11,12]. The right ventricular function is primarily affected in cases of primary organ failure; it is significantly more severe and more frequent than the left ventricle. Routine open-heart procedures including heart valve replacement, coronary artery bypass grafting or aneurysm surgery expose the heart to various periods of ischemia. Hypothermic storage suppresses basal metabolic rate and modifies signaling pathways towards expression of protective genes [13–21]. Nevertheless, energy utilization during ischemia continues at a reduced level even at 0 °C [22]. Heart storage has been significantly improved by various strategies, including cardioplegia, hypothermia, preconditioning and addition of various protective compounds to specifically formulated preservation solutions [8,19,22]. A number of preservation solutions have been studied and some were introduced into clinical practice in order to protect the heart during surgery (Custodiol; University of Wisconsin, UW, Celsior; *etc.*). Preservation solutions may include impermeants and osmotic agents to reduce edema and minimise cellular swelling (e.g., pentastarch, lactobionate, raffinose in UW; mannitol in Custodiol), buffering capacity against acidosis (histidine, phosphate), energy substrates (α-ketoglutarate; adenosine/ribose) to support energy status and antioxidants and inhibitors of ROS production [19–21].

In the case of coronary artery bypass surgery, graft vessels are harvested from the leg and forearm and stored during surgery until they are used for grafting. Therefore, these blood vessels are exposed to an ischemic period. In most cardiac surgical centers these blood vessels are stored in pure saline solutions, in some instances heparin is added [14].

In the current study we aimed to assess mitochondrial oxidative phosphorylation as an indicator of mitochondrial function and effects of cold ischemia. Mitochondrial function was measured in the left and right ventricles after 14 h of cold ischemia. In addition, the protective effect of cardioplegic/preservation solution (Custodiol^®^) on mitochondrial function was investigated and compared with simple saline solution. Additionally, mitochondrial function in various blood vessels including the ascending aorta, the left internal thoracic artery and the saphenous vein were investigated with regard to exposure to prolonged cold ischemia with and without using preservation solution. Here therefore we show significant benefit of Custodiol-HTK solution in myocardial preservation as compared with saline.

## 2. Results and Discussion

### 2.1. Analysis of Cold Ischemia Effects on Pig Myocardium

In our study we demonstrate that in controls, mitochondrial oxidative phosphorylation was similar in the left and right ventricle at the level of both mitochondrial complex I (with glutamate + malate), and complex II (with succinate). This can be important information since the usual strong requirements to use always very standardized biopsy samples regarding ventricle types may probably be avoided. Also, in controls (without cold ischemia), respiration data were not distinguished between the two solutions. Like previous findings using various heart cold ischemia models [21], 14 h of cold storage produced a decrease in mitochondrial function also in pig myocardium (up to about 60% of corresponding controls).

**Figure 1:**
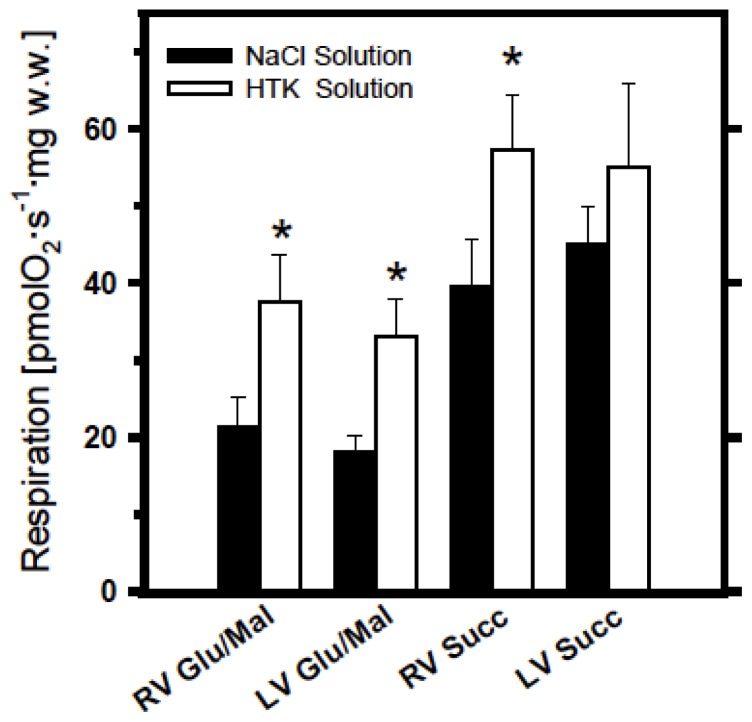
Respiratory activities of mitochondria in permeabilized myocardial fibers cryopreserved in liquid nitrogen using special DMSO containing preservation solution, see [2] after 14 h cold storage in either NaCl solution (**filled bars**) or in Bretschneider’s histidine-tryptophan-ketoglutarate (HTK) preservation solution (**empty bars**). Respiration was measured at 30 °C with glutamate + malate or with succinate + rotenone as described in Methods Section and expressed in pmols oxygen per second, per mg wet weight. Significantly higher respiration rates can be seen after cold ischemia in HTK solution (*p < 0.05).

Figure 1 demonstrates respiratory activities of mitochondria in permeabilized myocardial fibers isolated from the tissue after cold storage (14 h) in saline and Custodiol-HTK solutions. It can be seen that cold storage of myocardial samples prepared both from the right and left ventricles resulted in significantly better preservation of mitochondrial function in Custodiol-HTK than in simple saline solution (see Figure 1). This observation is valid for both glutamate + malate- and succinate + rotenone-supported respiration, however, differences between the two solutions is more evident for complex I substrates, suggesting that cold ischemia produces more damaging effects on complex I than on complex II. Moreover, higher mitochondrial respiration rates measured with mitochondrial substrate succinate + rotenone as compared with glutamate + malate may also reflect higher susceptibility of complex I to storage.

### 2.2. Analysis of the Effects of Cold Ischemia on Mitochondrial Function in Various Pig Blood Vessels

**Figure 2:**
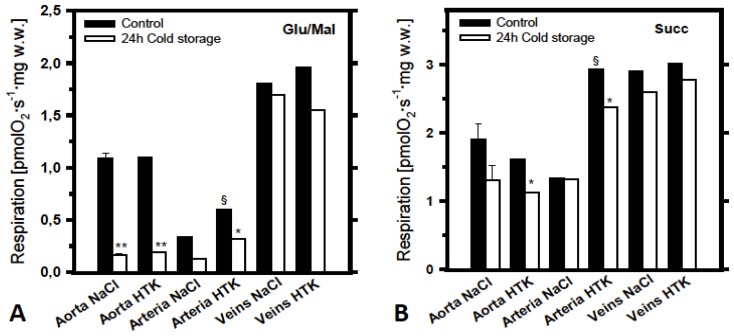
Respiratory activities of mitochondria in permeabilized aorta, arterial or venous fibers (cryopreserved in DMSO containing preservation solution, see reference [2]). Mitochondrial respiration of corresponding controls (without cold ischemia, **filled bars**) is compared with mitochondrial respiratory activities after 24 h cold storage (**empty bars**) in either saline NaCl solution or in Bretschneider’s histidine-tryptophan-ketoglutarate (HTK) preservation solution. As in Figure 1, respiration was measured at 30°C with glutamate + malate (**A**) or with succinate + rotenone (**B**) as mitochondrial substrates and expressed in pmols oxygen per second, per mg wet weight. *: *p* < 0.05 and **: *p* < 0.01 as compared with controls; §: *p* < 0.05 as compared with corresponding saline NaCl groups.

Overall, all vessel samples including aorta, arteries and veins showed 20–30 fold lower mitochondrial activity compared with myocardial samples (compare absolute rate values in Figures 1 and 2).

Figure 2 shows effects of 24 h cold storage on mitochondrial respiration in these blood vessels. Measurements with complex I and II substrates glutamate + malate and succinate + rotenone are shown separately (A and B). As was observed in the heart, cold ischemia significantly reduces mitochondrial function in aorta; a much lower effect can be seen in arteries (Figure 2A). Samples of saphenous veins showed a high grade of resistance against ischemia. Even after 24 h of cold ischemia no effect on mitochondrial respiration was found regardless of the storage solution (Custodiol or saline). Also, it can be seen that, like the results obtained from the pig myocardium experiments, respiration with succinate was significantly higher and the effects of cold storage measured with succinate was significantly lower than that with glutamate + malate. In contrast to the myocardium, aorta and veins demonstrated equal mitochondrial function after 24 h of cold storage in both Custodiol-HTK and saline solutions. However, some superiority of Custodiol-HTK for preservation of mitochondrial function was observed in arteries using both succinate and glutamate + malate as mitochondrial substrates (Figure 2A,B).

### 2.3. Discussion

In the present study using a pig model we investigated the effects of two solutions (saline and Custodiol-HTK) on cold ischemia-reperfusion injury with respect to mitochondrial respiration through different segments of the electron transport system. Mitochondrial damage during ischemia including cold ischemia is the key event leading to cell death on a cellular level and organ dysfunction on a larger scale [8,21–24]. Our findings suggest thus an association between mitochondrial damage and functional capacity of the organ. The results reported herein describe analytical method that allows various measurements of mitochondrial activity *in situ* in permeabilized muscles and tissues without isolation of mitochondria [1]. Importantly, this approach allows the analysis of mitochondria to be performed within an integrated cellular system, preserving essential interactions with the cytoskeleton, nucleus and endoplasmic reticulum avoiding many artefacts of mitochondrial isolation. Although this method is not able to separately investigate mitochondrial subpopulations, it provides a novel sensitive approach for monitoring mitochondria-related injuries in small biopsies and may find use in the development and evaluation of newly designed preservation solutions. In accordance with previous data [25], we demonstrate here that Custodiol-HTK preservation solution can be effective for the protection of mitochondrial function also in the pig myocardium when exposed to cold ischemia.

Right and left ventricular myocardium showed no difference at mitochondrial level at baseline and the mitochondrial function similarly declined by the same amount in both ventricles when exposed to prolonged cold ischemia. In the clinical setting of heart transplantation it is often the case that the right ventricle recovers slower from ischemia than the left ventricle. Weaning from cardiopulmonary bypass may be difficult due to right ventricular failure. However, this phenomenon can obviously not be explained by the effects of ischemia on the energy metabolism, since mitochondrial function of right and left ventricular myocardium did not differ in our study. An alternative explanation might be the different geometry of the right ventricle and the fact that the right ventricular wall in general is thinner than the left ventricular wall and therefore more vulnerable to stunning. Furthermore, pulmonary hypertension in heart-transplant recipients may significantly impair the right ventricle of the donor heart, which is not adapted to such high pressure-levels. This study thereby contributes to the existing but incongruent knowledge of the superiority of preservation solution over purely saline solutions [25]. Also, investigation of the mitochondrial damage together with the analysis of the associated changes in the respiratory chain give a better insight into the mechanism of damage resulting in the impaired function [7,9,25,26].

Blood vessels in general have much lower mitochondrial capacities compared with myocardium (compare respiration rates shown in Figures 1 and 2). This was an expected finding due to the fact that the myocardial muscle contains a very high amount of very active mitochondria (about 30% of cardiomyocyte volume). Interestingly, in some blood vessels like veins this low energetic level seems to be rather stable during cold ischemia, reflecting different modes of their energy metabolism. Quite opposite to the myocardium, even prolonged ischemia times of 24 h resulted in nearly no effect in venous grafts, in contrast to similarly strong effects in aorta and some effects in arterial grafts (Figure 2A). Notably, much stronger effects were observed in relation to complex I damage than that to complex II (Figure 2A *vs.* Figure 2B), pointing to similar higher vulnerability of complex I to ischemia also in blood vessels. This is consistent with the previous findings that usually complex I can be especially sensitive to ischemia/reperfusion injury, in contrast to a relatively high stability of respiratory complexes II and IV [7]. However, no significant differences were found between Custodiol-HTK and saline solution in blood vessels. When harvesting blood vessels for coronary artery bypass surgery, most centers usually use pure saline solution. Attempts to use other solutions for better preservation of these grafts by aiming to improve long-term patency rates failed to show significant clinical improvements.

#### Limitations

One essential limitation of this study is that we did not use freshly isolated preparations. Instead we used frozen liquid nitrogen samples, where mitochondrial function was cryopreserved in special DMSO-contained solution. This still may have some (we believe minimal) influence on mitochondrial respiratory function. Significant advantages of this approach however, as mentioned above, is that many samples can be collected simultaneously and measured later during several days. Also, due to technical problems, we did not analyze cardiac function and effects of reperfusion after cold ischemia and heart transplantation. We now provide metabolic evidence that the short intraoperative ischemic periods (10–24 h) probably have no effect on mitochondrial function in blood vessels. Therefore, in contrast to the heart and some other high-energy metabolism-dependent organs, special storage solutions for blood vessels are of questionable benefit in this application.

## 3. Experimental Section

### 3.1. Animals

Male landrace pigs weighing between 60 and 80 kg served as study animals. All animals received human care in compliance with the “Principles of Laboratory Animal Care” (NIH Publication No. 86-23, revised 1985, Bethesda, MD, USA) as well as with the Austrian law. The animals were premedicated with intramuscular ketamine (10 mg/kg), intubated, and ventilated at 10 mL/kg with isoflurane (1%–3%) and oxygen. During surgery anaesthesia was maintained with propofol 1% (Fresenius Kabi Austria GmbH, Graz, Austria) and piritriamid 75 mg/kg/h (Dipidolor, Janssen-mCilag Pharma Gmbh, Vienna, Austria).

The heart, the ascending aorta, internal mammary arteries and saphenous veins were harvested and immediately prepared for analyses or exposed to cold ischemia at 4 °C in either saline or Custodiol (HTK) solution. Myocardial samples were stored for 14 h. Respirometry of blood vessels was performed at two time points, immediately after harvesting and after 24 h of cold storage.

### 3.2. Analysis of Mitochondrial Function

We used an *in situ* method for mitochondrial respirometry of cryopreserved and permeabilized muscle tissues as described previously [2]. Myocardial (ventricle) or vessel tissues were dissected according to previously published method [1,9] and cryopreserved in liquid nitrogen using a special dimethyl sulfoxide (DMSO) containing preservation solution as described in [20]. Since one respiration measurement requires about 1 h, sample analysis was performed sequentially during the next several days. This reflects the most essential advantage of cryopreservation approach in mitochondrial analysis. Of note, the time of the contact with DMSO was minimized to 3–5 s. After thawing, preparations were immediately washed with the medium for respiration measurement, which does not contain DMSO [20]. Mitochondrial respiratory function was measured in saponin (50 μg/mL) permeabilized muscle fibers by high-resolution respirometry at 30 °C, using two-channel titration-injection respirometers (Oroboros Oxygraph, Innsbruck, Austria, [1,21]) and expressed in pmols oxygen per second, per mg wet weight. The respiration medium consisted of 110 mM sucrose, 60 mM K-lactobionate, 0.5 mM ethylene glycol-bis(2-aminoethylether)-*N*,*N*,*N′*,*N′*-tetraacetic acid (EGTA), 1 g/L bovine serum albumin (BSA) essentially fatty acid free, 3 mM MgCl_2_, 20 mM taurine, 10 mM KH_2_PO_4_, 20 mM 4-(2-hydroxyethyl)piperazine-1-ethanesulfonic acid (HEPES), pH 7.1. DatLab software (Oroboros Instruments, Innsbruck, Austria) was used for data acquisition and analysis. Respiration was stimulated by 1 mM adenosine diphosphate (ADP) (maximum rate, state 3) and measured with 10 mM glutamate and 5 mM malate (substrates for complex I of the mitochondrial respiratory chain) or using 10 mM succinate and 0.5 μM rotenone (substrate for complex II). Typically, the stimulatory effects of ADP (state3/state2 ratios) in cryopreserved myocardial preparations were in range 3.4–4.7 in the presence of complex I substrates glutamate + malate.

### 3.3. Storage Solutions and Chemicals

Filtered (Millipore, Billerica, MA, USA; 0.22 μm) saline and Bretschneider’s histidine-tryptophan-ketoglutarate (HTK; Custodiol^®^ Dr. Koehler Chemie GMBH, Alsbach-Haenlein, Germany) solutions were used for organ flush and cold storage. EGTA, BSA, HEPES and ADP were purchased from “Sigma” (Vienna, Austria).

## 4. Conclusions

In summary, HTK-Custodiol solution offers significant protection of myocardial mitochondria against cold ischemic injury and can be used as efficient preservation solution in organ transplantation. High-resolution respirometry analysis of mitochondrial function in permeabilized preparations can be used as a suitable method for the assessment of cold ischemic injury in various tissues including heart and also various blood vessels.
